# Awareness and attitudes toward traditional and complementary medicine among community-dwelling individuals in Türkiye: a cross-sectional online survey

**DOI:** 10.1186/s12906-026-05480-6

**Published:** 2026-07-22

**Authors:** Akın Süzer, Sinem Yenil-Kocabay, Nevriye Ünal-Süzer, Nihal Büker

**Affiliations:** 1 Therapy and Rehabilitation Department, Burdur Mehmet Akif Ersoy University Burdur Vocational School of Health Services, Burdur, Türkiye; 2 Faculty of Physiotherapy and Rehabilitation, Pamukkale University, Denizli, Türkiye; 3 Medical Services and Techniques Department, Burdur Mehmet Akif Ersoy University, Burdur Vocational School of Health Services, Burdur, Türkiye

**Keywords:** Traditional medicine, Complementary medicine, Attitude, Awareness, Community-Dwelling, Online Survey

## Abstract

**Background:**

As traditional and complementary medicine (T&CM) becomes increasingly widespread, assessing the awareness and attitudes of community-dwelling individuals is essential to understand its current status and promote its rational use. Therefore, this study aims to evaluate the awareness levels and attitudes of community-dwelling individuals in Türkiye regarding T&CM practices.

**Methods:**

This cross-sectional online survey included 1375 individuals residing in the community across Türkiye. Data were collected through a structured questionnaire via Google Forms™, including items on sociodemographic characteristics, awareness, opinions, and experiences with T&CM. Attitudes were assessed using the Complementary, Alternative and Conventional Medicine Attitude Scale (CACMAS). Data were analysed using IBM SPSS Statistics 25. Multiple linear regression analysis was performed to identify independent predictors of attitudes.

**Results:**

The most recognized T&CM practices were massage (91.13%), wet cupping (84.51%), and medicinal leech therapy (80.29%). Overall, 31.78% received T&CM information from healthcare professionals, 22.25% used it, and 60.73% recommended it. The mean CACMAS score was 108.78 ± 19.51, differing significantly by age (*p* = 0.004). T&CM usage varied by age, marital status (both *p* < 0.001), education (*p* = 0.006), and chronic disease (*p* = 0.004). Receiving T&CM information from professionals was associated with age (*p* = 0.029), education (*p* = 0.007), and perceived health (*p* = 0.023). Multiple linear regression revealed that increasing age (*p* = 0.006), female gender (*p* = 0.011), marital status (*p* = 0.049), and previous T&CM experience (*p* < 0.001) independently predicted favorable attitudes, whereas education (*p* = 0.945) and chronic disease (*p* = 0.218) lost significance.

**Conclusion:**

Although T&CM usage rates are relatively low, community-dwelling individuals in Türkiye exhibit generally favorable attitudes. These attitudes are independently shaped by specific sociodemographic factors, such as age and gender, rather than education or chronic disease. These findings may provide the basis for the rational use of T&CM and highlight the importance of further studies that take cultural and regional diversity into account.

**Supplementary Information:**

The online version contains supplementary material available at https://doi.org/10.1186/s12906-026-05480-6.

## Background

Traditional and complementary medicine (T&CM) has been widely used globally for health maintenance and the prevention and management of diseases [1, 2]. However, the increasing popularity of T&CM has raised significant public health and safety concerns [3]. Issues such as the use of unregulated products, potential herb-drug interactions, and the frequent failure of patients to disclose T&CM use to their conventional healthcare providers pose considerable risks to patient safety [3, 4]. Therefore, understanding the public’s awareness and attitudes is critical to prevent misuse and ensure safe integration into the healthcare system [5, 6].

Establishing a knowledge base for the active management of T&CM through appropriate national policies is one of the World Health Organization’s (WHO) strategic objectives [7]. In this context, to regulate T&CM practices and ensure their safe application within the local context of Türkiye, the Turkish Ministry of Health published the T&CM Regulation in 2014 [8]. This regulation established a legal framework for 15 modalities, including acupuncture, apitherapy, phytotherapy, hypnosis, leech therapy, homoeopathy, chiropractic, wet cupping therapy (hijama), larvae therapy, mesotherapy, prolotherapy, osteopathy, ozone therapy, reflexology, and music therapy, determining by whom and where they can be performed [8]. Despite these regulations, T&CM practices have deep cultural roots and are still widely utilized among the general population in Türkiye [9, 10].

In addition, in line with its strategic goals, WHO emphasises the need to analyse why and when people use T&CM, what the benefits of T&CM are, and by whom they are provided [7]. In the research on this subject, it is stated that the level of awareness and attitude towards T&CM is very important in order to better understand the current situation of T&CM and the motivations of patients to use T&CM [5, 11]. While studies investigating the level of awareness and attitude of different populations such as medical faculty students [12], primary care patients [9], and oncology patients [13] in Türkiye regarding T&CM practices are available in the literature, the number of studies examining the awareness and attitude of individuals residing in the community regarding T&CM practices remains limited.

The aim of this study is to investigate the level of awareness and attitudes of individuals residing in the community about T&CM practices.

## Methods

### Study design and setting

This study was designed as an online survey-based descriptive cross-sectional study. Ethical approval was obtained from Pamukkale University Non-Invasive Clinical Research Ethics Committee (number: E-60116787-020-569460, date: 22.08.2024). This study is reported in accordance with the Strengthening the Reporting of Observational Studies in Epidemiology (STROBE) guidelines. In this study, data were collected between September and December 2024. The link to the online version of the data collection instruments, which had been prepared in advance using Google Forms™, was distributed to potential participants via WhatsApp™ messaging groups. Individuals who were accessible to the researchers and met the inclusion criteria were invited to participate voluntarily. The survey link included the contact details of the relevant researcher (Akın SÜZER) and an informed consent statement. Before accessing the questionnaire, participants were required to read the consent form and indicate their agreement by selecting the option “I agree to participate in this study voluntarily without any pressure or coercion.” Only those who provided this consent were included in the study. The survey was open to the general population and not directed toward any specific subgroup such as healthcare professionals, students, or patients. The link was shared broadly through general WhatsApp™ messaging groups and personal networks, allowing participation from community-dwelling individuals in Türkiye who met the inclusion criteria. Participants were recruited from different regions of Türkiye through online communication networks; however, the study was not designed as a nationally representative survey and no region-based sampling strategy was applied.

Participants’ names, e-mail addresses, IP addresses, and other personally identifiable information were not collected by the researchers. All responses were recorded anonymously, and no data accessible to the research team could be linked to individual participants. Although Google Forms™ was configured to limit submissions to one response per Google account in order to reduce duplicate entries, the researchers did not have access to participants’ Google account information. The dataset was stored in a password-protected electronic file accessible only to the research team.

### Participants

Participants were recruited using convenience sampling, a non-probability sampling method based on participants being accessible to researchers and voluntarily agreeing to participate. As this study employed an online survey-based design, convenience sampling was appropriate as it enabled researchers to quickly and practically collect data from individuals who were accessible and willing to participate. A total of 1375 individuals living in Türkiye, between the ages of 18–80, who can speak and understand Turkish were included in the study. The inclusion criteria were: being between 18 and 80 years of age, living in Türkiye, being able to read and understand Turkish, having access to the online survey platform, and voluntarily agreeing to participate in the study. Individuals younger than 18 years of age and questionnaires that were incomplete were excluded from the study. Participation in the study was voluntary, and participants did not receive any incentive.

**Fig. 1:**
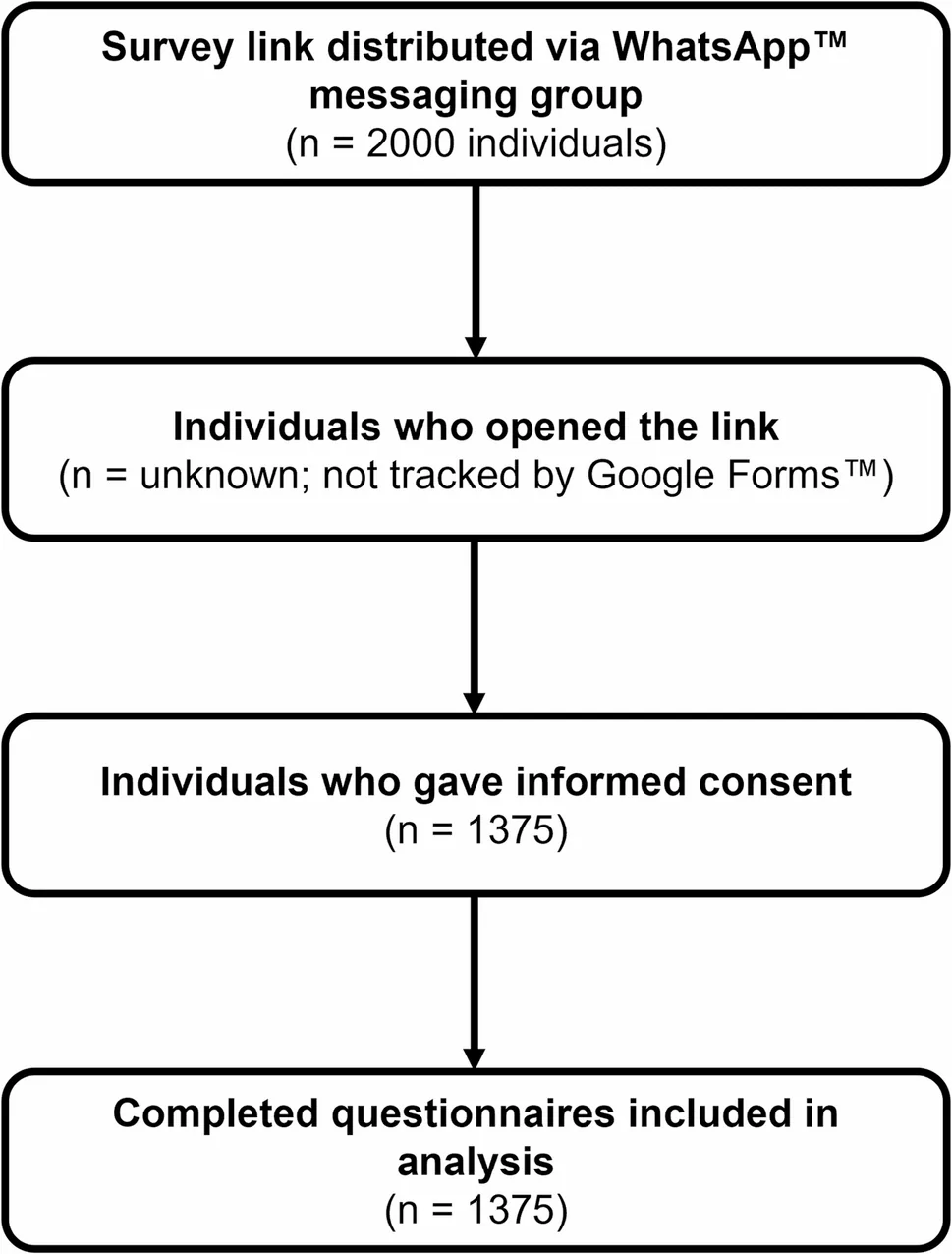
Flow diagram of participant recruitment and survey response

The survey link was distributed to approximately 2000 individuals via WhatsApp™ messaging groups. A total of 1375 participants completed the questionnaire in full. As the Google Forms™ platform did not collect IP addresses or use cookies to track visitors, unique site visitors were not identified. Therefore, view and participation rates could not be calculated based on unique visitors. However, based on the number of distributed links, the estimated participation rate was approximately 68.8% (1375/2000). The completion rate was 100% among those who participated, as only fully completed questionnaires were included in the analysis (Fig. 1).

### Data collection instruments

Data collection instruments consisted of a descriptive information form, an awareness level form about T&CM practices, an opinion and experience form about T&CM, and the Complementary, Alternative and Conventional Medicine Attitude Scale (CACMAS). Completeness checks were performed automatically by the Google Forms™ platform. Items that were mandatory were defined as “required,” which prevented participants from submitting the questionnaire unless all mandatory fields were completed. In addition, each question included a “no” or “I have no awareness” option to allow for non-response when appropriate. No additional manual or JavaScript-based data validation was implemented. Respondents were able to review and change their answers before submitting the questionnaire. The Google Forms™ platform allowed participants to navigate back to previous questions and modify their responses as needed. However, once the form was submitted, participants were not able to revise or resubmit their answers.

Sociodemographic characteristics of the participants including age, gender, education level, marital status, chronic disease and perceived health were determined through the descriptive information form. The level of awareness of the participants about 25 different T&CM practices was determined as awareness or no awareness through the awareness level form about T&CM practices. In this study, awareness refers to participants’ self-reported recognition or familiarity with the names of T&CM practices and does not represent objective clinical knowledge, evidence-based understanding, or awareness of the scientific evidence regarding these practices. Based on the literature review [4, 8, 14], the researchers developed a four-item form to assess participants’ opinions and experiences regarding T&CM practices. These items were developed solely to obtain descriptive information regarding participants’ opinions and experiences related to T&CM practices and were not intended to constitute a psychometrically validated scale. The items were designed as dichotomous (Yes / No) questions. To ensure clarity and face validity, the items were reviewed by three experts in physiotherapy, and minor wording adjustments were made based on their feedback.

Participants’ attitudes towards T&CM were assessed using the validated Complementary, Alternative and Conventional Medicine Attitude Scale (CACMAS). CACMAS consists of 27 items designed to assess attitudes and beliefs regarding T&CM approaches. CACMAS, which has no cut-off value, has three sub-dimensions: 1. philosophical congruence with complementary and alternative medicine (8 items), 2. dissatisfaction with conventional medicine (10 items), and 3. holistic balance (9 items). Each item in CACMAS is scored on a 7-point Likert Scale ranging from 1 (“strongly disagree”) to 7 (“strongly agree”). As CACMAS does not have a defined cut-off value, it does not categorically classify attitudes as ‘positive’ or ‘negative’. Instead, higher total scores on the scale indicate more favorable attitudes towards T&CM [6]. The Turkish validity and reliability study of CACMAS was conducted in 2018 and demonstrated acceptable psychometric properties. With a Cronbach’s alpha value of 0.808, the Turkish version of CACMAS is reported to be highly reliable [15].

### Study size

In this study, the minimum required sample size was calculated based on the study conducted by Öztürk et al. (2022) using CACMAS on a different sample in the Turkish population [16]. The calculation, performed with a 5% margin of error, 95% confidence interval, 80% power, and 0.05 significance level, determined that a minimum of 986 participants were required. The G*Power 3.1 software was used to calculate the sample size.

### Statistical analysis

Data was analysed using IBM SPSS Statistics 25 (IBM Corp., Armonk, New York, USA). Continuous variables were presented as mean ± standard deviation and categorical variables as number of units (n) and percentage (%). The normality of the data distribution was assessed using the Shapiro-Wilk test. Pearson’s Chi-Squared test was used for the comparison of categorical variables. For the comparison of two independent groups, the Independent Samples T-test was used when parametric assumptions were met, and the Mann-Whitney U test was used when nonparametric assumptions were met. For the comparison of three independent groups, the One-Way ANOVA test was used under parametric assumptions, and the Kruskal-Wallis H test was used under nonparametric assumptions. Multiple linear regression analysis was performed to identify factors independently associated with CACMAS total score. Sociodemographic characteristics (age, gender, education level, marital status), health-related factors (chronic disease and perceived health status), and T&CM-related variables (obtaining information from healthcare professionals, previous T&CM use, family use of T&CM, and recommending T&CM) were entered simultaneously into the regression model. Multicollinearity diagnostics were assessed before regression analysis. In all analyses, *p* < 0.05 was considered statistically significant.

## Results

In this study, 1375 people residing in the community participated. The mean age of the participants was 34.14 ± 14.24 years. Of the participants, 61.45% were female and 68.80% had a university education or higher. The sociodemographic characteristics of the participants are presented in Table 1.

**Table 1:** Sociodemographic characteristics of the participants

		n	%
Age	18–24	551	40.07
	25–44	443	32.22
	≥ 45	381	27.71
Gender	Female	845	61.45
	Male	530	38.55
Education level	High school and below	429	31.20
	University and above	946	68.80
Marital status	Single	775	56.36
	Married	600	43.64
Chronic disease	Yes	227	16.51
	No	1148	83.49
Perceived health	Very good / Good	891	64.80
	Medium	450	32.73
	Bad / Very bad	34	2.47

The T&CM practices with the highest level of awareness among the participants were massage [*n* = 1253 (91.13%)], wet cupping therapy (hijama) [*n* = 1162 (84.51%)], medicinal leech therapy (hirudotherapy) [*n* = 1104 (80.29%)], yoga [*n* = 1018 (74.04%)], and meditation [*n* = 993 (72.22%)]. The T&CM practices with the lowest awareness were ayurveda [*n* = 112 (8.15%)], acupressure [*n* = 141 (10.26%)], homeopathy [*n* = 168 (12.22%)], reiki [*n* = 197 (14.33%)], and relaxation [*n* = 210 (15.27%)]. The awareness levels of the participants regarding T&CM practices are presented in Table 2.

**Table 2:** Participants’ awareness level towards T&CM applications

	No Awareness (*n* = 1375)		Awareness (*n* = 1375)	
	*n*	%	*n*	%
Acupressure	1234	89.75	141	10.26
Acupuncture	451	32.80	924	67.20
Ayurveda	1263	91.86	112	8.15
Chiropractic	863	62.76	512	37.24
Cupping Therapy	573	41.67	802	58.33
Dry Needling	879	63.93	496	36.07
Energy Medicine (Bioenergy)	682	49.60	693	50.40
Herbal Medicine (Phytotherapy)	478	34.76	897	65.24
Homeopathy	1207	87.78	168	12.22
Hydrotherapy	922	67.06	453	32.95
Hypnosis	512	37.24	863	62.76
Lifestyle Diet	693	50.40	682	49.60
Massage	122	8.87	1253	91.13
Medicinal Leech Therapy (Hirudotherapy)	271	19.71	1104	80.29
Meditation	382	27.78	993	72.22
Music Therapy	584	42.47	791	57.53
Neural Therapy	1145	83.27	230	16.73
Nutritional Therapy	629	45.75	746	54.26
Reflexology	1093	79.49	282	20.51
Reiki	1178	85.67	197	14.33
Relaxation	1165	84.73	210	15.27
Tai Chi	1110	80.73	265	19.27
Therapeutic Touch	950	69.09	425	30.91
Wet Cupping Therapy (Hijama)	213	15.49	1162	84.51
Yoga	357	25.96	1018	74.04

Awareness: Refers to the participant selecting the “I know” option for the respective Traditional and Complementary Medicine (T&CM) practice, indicating self-reported recognition or familiarity. No Awareness: Refers to the participant selecting the “I don’t know” option for the respective T&CM practice

**Fig. 2:**
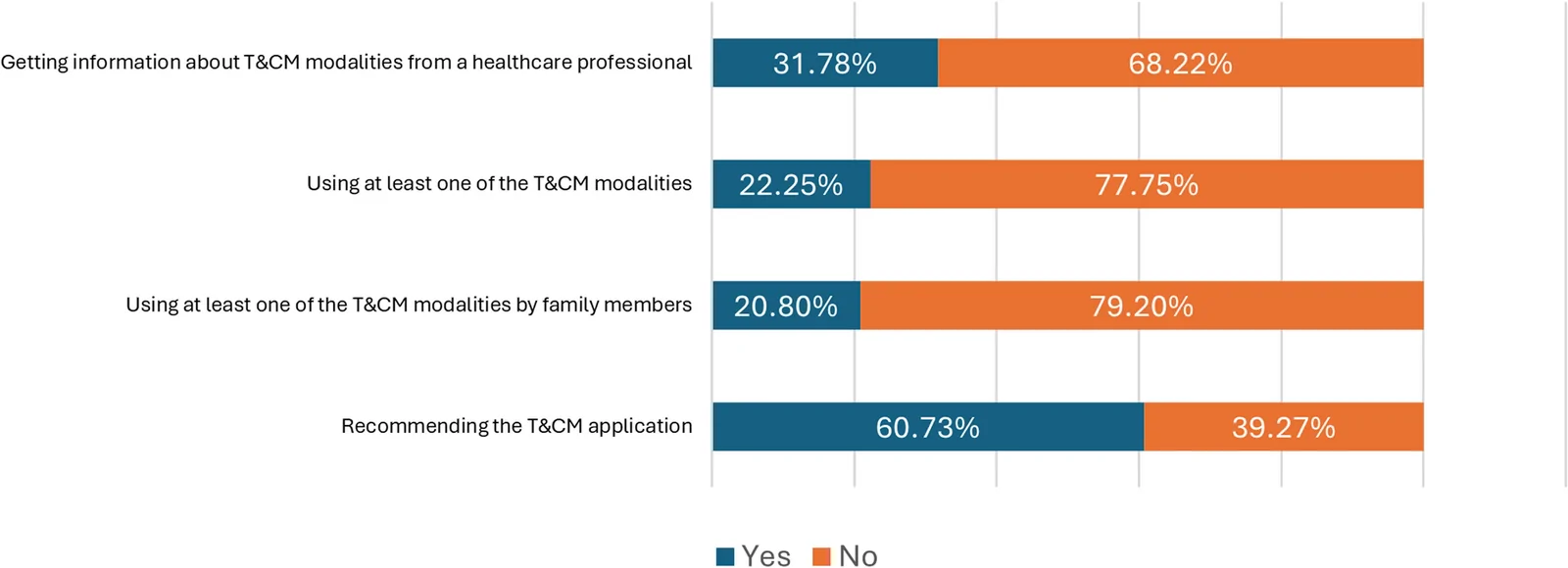
Participants’ opinions and experiences towards T&CM applications

Overall, 31.78% (*n* = 437) of the participants had received information about T&CM from a health professional. Furthermore, 22.25% (*n* = 306) of the participants had used a T&CM modality at least once, and 60.73% (*n* = 835) stated they would recommend T&CM practices to others. The opinions and experiences of the participants regarding T&CM practices are presented in Fig. 2.

*Regarding the attitudes toward T&CM practices*,* the mean CACMAS total score was 108.78 ± 19.51*. The CACMAS total and sub-dimension scores of the participants are given in Table 3.

**Table 3:** Participants’ attitudes towards T&CM applications

	X ± SD
CACMAS total score	108.78 ± 19.514
Philosophical congruence with complementary and alternative medicine	33.805 ± 9.114
Dissatisfaction with conventional medicine	30.770 ± 10.593
Holistic balance	44.205 ± 9.180

In the bivariate analyses, the CACMAS total score showed a statistically significant difference between age groups (*p* = 0.004). The rate of having used a T&CM modality at least once showed a statistically significant difference according to age group (*p* < 0.001), education level (*p* = 0.006), marital status (*p* < 0.001), and the presence/absence of a chronic disease (*p* = 0.004). In addition, receiving information about T&CM modalities from a health professional showed statistically significant differences according to age group (*p* = 0.029), educational status (*p* = 0.007), and perceived health status (*p* = 0.023) (Table 4).

**Table 4:** Comparison of participants’ attitudes, opinions and experiences towards T&CM applications based on sociodemographic characteristics

		CACMAS Total Score X ± SD	Getting information about T&CM modalities from a healthcare professional			Using at least one of the T&CM modalities		
			Yes *n* (%)		No *n* (%)	Yes *n* (%)		No *n* (%)
Age	18–24	106.64 ± 18.63	153 (27.77)		398 (72.23)	66 (11.98)		485 (88.02)
	25–44	110.09 ± 20.07	156 (35.21)		287 (64.79)	114 (25.73)		329 (74.27)
	≥ 45	110.36 ± 19.82	128 (33.60)		253 (66.40)	126 (33.07)		255 (66.93)
		***p*** = 0.004*		p = 0.029*			***p*** < 0.001*	
Gender	Female	109.98 ± 19.07	274 (32.43)		571 (67.57)	189 (22.37)		656 (77.63)
	Male	106.87 ± 20.06	163 (30.75)		367 (69.25)	117 (22.08)		413 (77.92)
		*p* = 0.366		p = 0.517			*p = 0.899*	
Education level	High school and below	109.40 ± 18.92	122 (27.91)		315 (72.09)	109 (26.45)		303 (73.55)
	University and above	108.50 ± 19.77	290 (33.30)		631 (66.70)	197 (20.82)		749 (79.18)
		*p* = 0.699		p = 0.007*			p = 0.006*	
Marital status	Single	108.07 ± 19.81	238 (30.71)		537 (69.29)	118 (15.23)		657 (84.77)
	Married	109.69 ± 19.08	199 (33.17)		401 (66.83)	188 (31.33)		412 (68.67)
		*p* = 0.112		p = 0.332			p < 0.001*	
Chronic disease	Yes	108.94 ± 18.27	82 (36.12)		145 (63.88)	67 (29.52)		160 (70.48)
	No	108.75 ± 19.75	355 (30.92)		793 (69.08)	239 (20.82)		909 (79.18)
		*p* = 0.071		*p* = 0.124			p = 0.004*	
Perceived health	Very good / Good	108.28 ± 19.69	296 (33.22)		595 (66.78)	202 (22.67)		689 (77.33)
	Medium	110.12 ± 19.31	137 (30.44)		313 (69.56)	99 (22.00)		351 (78.00)
	Bad / Very bad	104.24 ± 15.79	4 (11.76)		30 (88.24)	5 (14.71)		29 (85.29)
		*p* = 0.961		p = 0.023*			*p* = 0.542	

*CACMAS* Complementary and Alternative Medicine Consciousness and Attitude Scale; *T&CM* traditional and complementarymedicine; *X±* mean; *SD* standard deviation; *n* number of participants; %, percentage

*p* values represent comparisons between the corresponding sociodemographic groups

Statistically significant *p* values are shown in bold and marked with an asterisk (*p* < 0.05)

To identify the independent factors associated with the CACMAS total score, a multiple linear regression analysis was conducted (Table 5). The overall regression model was statistically significant (R² = 0.095, Adjusted R² = 0.087, *p* < 0.001). The analysis revealed that older age (25–44 years: = 0.084, *p* = 0.018; 45 years: = 0.116, *p* = 0.006), female sex (= 0.066, *p* = 0.011), previous T&CM use (= 0.110, *p* < 0.001), and recommending T&CM to others (= 0.195, *p* < 0.001) were independently and significantly associated with higher CACMAS scores (i.e., more favorable attitudes towards T&CM). Conversely, being married was independently associated with a lower CACMAS score ( = -0.076, *p* = 0.049).

**Table 5:** Multiple linear regression analysis of factors associated with CACMAS total score

	B	SE	β	*p*
Age (25–44 vs. 18–24)	**3.524**	**1.491**	**0.084**	**0.018**
Age (≥ 45 vs. 18–24)	**5.099**	**1.871**	**0.116**	**0.006**
Female sex (vs. male)	**2.662**	**1.049**	**0.066**	**0.011**
University and above (vs. high school and below)	-0.081	1.189	-0.002	0.945
Married (vs. single)	**-2.997**	**1.520**	**-0.076**	**0.049**
Chronic disease (yes vs. no)	-1.802	1.462	-0.034	0.218
Perceived health: Medium (vs. bad/very bad)	5.076	3.287	0.121	0.123
Perceived health: Good/very good (vs. bad/very bad)	2.996	3.250	0.073	0.357
Information from healthcare professionals (yes vs. no)	0.321	1.220	0.008	0.793
Previous T&CM use (yes vs. no)	**5.190**	**1.468**	**0.110**	**< 0.001**
Family use of T&CM (yes vs. no)	2.304	1.400	0.048	0.100
Recommending T&CM (yes vs. no)	**7.833**	**1.161**	**0.195**	**< 0.001**
R²	**0.095**			
Adjusted R²	**0.087**			
Model p	**< 0.001**			

B, unstandardized regression coefficient; SE, standard error; β, standardized regression coefficient; R², coefficient of determination. Statistically significant predictors are shown in bold (*p* < 0.05). Multiple linear regression analysis was performed to identify factors independently associated with CACMAS total score. Sociodemographic characteristics (age, gender, education level, marital status), health-related factors (chronic disease and perceived health status), and T&CM-related variables (obtaining information from healthcare professionals, previous T&CM use, family use of T&CM, and recommending T&CM) were entered simultaneously into the regression model. Multicollinearity diagnostics were assessed before regression analysis. R² and adjusted R² values indicate the proportion of variance in CACMAS total score explained by the model. Reference categories were: age 18–24 years, male sex, high school and below education, single marital status, no chronic disease, bad/very bad perceived health, no information from healthcare professionals, no previous T&CM use, no family use of T&CM, and not recommending T&CM

## Discussion

In this study, which investigated the awareness levels and attitudes of community-dwelling individuals in Türkiye regarding T&CM practices, it was determined that the T&CM modalities with the highest awareness among participants were massage, wet cupping therapy (hijama), medicinal leech therapy, yoga, and meditation. In addition, it was observed that a substantial majority of the participants recommended T&CM practices to others. Overall, the participants exhibited generally favorable attitudes towards T&CM practices. Furthermore, it was also found that these attitudes, opinions, and experiences regarding T&CM practices were independently predicted by specific sociodemographic characteristics and previous personal experiences.

In this study evaluating the awareness and attitudes of community-dwelling individuals in Türkiye regarding traditional and complementary medicine (T&CM) practices, we determined that participants exhibited generally more favorable attitudes towards these modalities, as indicated by their overall CACMAS scores. Concurrently, the practices with the highest awareness levels were massage, wet cupping therapy (hijama), medicinal leech therapy, yoga, and meditation. This widespread acceptance indicates that favorable dispositions toward T&CM practices are not confined to specific clinical settings but are deeply rooted and highly prevalent among the general public. While a substantial portion of the existing literature has predominantly focused on individuals directly related to the health sector, such as healthcare professionals and medical students [4, 12], there is also a growing body of evidence exploring the public and patient perspective [8, 9]. Our results demonstrate that the favorable trends observed in specific professional or academic subgroups are highly compatible with those of the broader community. In this context, contributing to the existing community-based research provides a much-needed broader perspective for a more comprehensive understanding of the multifaceted factors shaping societal attitudes toward T&CM.

Furthermore, our study highlighted that alongside globally popular mind-body practices like massage and yoga, traditional methods such as wet cupping therapy (hijama) and medicinal leech therapy share the highest awareness levels among the participants. This finding suggests a dual structure in the community’s approach to T&CM, where culturally embedded therapies appear to coexist with modern wellness practices, reflecting the strong local context of healthcare-seeking behaviors in Türkiye. The existing literature supports the influence of cultural and historical backgrounds on T&CM preferences, as other studies have similarly noted that methods with deep regional roots, such as cupping and leech therapy, are frequently recognized and sought by Turkish patients [9, 10]. Recognizing this dual awareness profile demonstrates that national T&CM regulations might benefit from being culturally sensitive, simultaneously accommodating global standards for mind-body practices while maintaining rigorous, safe, and culturally respectful frameworks for traditional practices like hijama.

In our study, despite the generally favorable attitudes towards T&CM, the actual rate of having used a T&CM modality at least once was found to be relatively low. This discrepancy suggests that for the Turkish community, T&CM serves primarily as an optional, complementary approach rather than a fundamental necessity for basic healthcare. When compared to the literature, this rate is considerably lower than those reported in developing countries, such as Ethiopia, where T&CM utilization can reach up to 80% due to factors like affordability, easy accessibility, and limited conventional medical resources [7, 14]. Considering that Türkiye has made progress in increasing access to modern conventional health services [17], the discrepancy in usage rates might be related to the robust accessibility of the modern healthcare infrastructure in Türkiye. This contextual difference highlights that as a country’s conventional healthcare system becomes more accessible and comprehensive, public reliance on T&CM may shift from being a necessity-driven primary care substitute to a choice-driven complementary wellness practice.

A striking finding regarding participants’ opinions and experiences was the discrepancy between actual use and peer recommendation: although only 22.25% of the participants had used a T&CM modality, a substantial majority (60.73%) stated they would recommend T&CM practices to others. This disconnect suggests that T&CM information often spreads through informal, word-of-mouth channels rather than reliable medical sources. Such unsupervised recommendations can pose a significant public health challenge, as individuals may erroneously assume that “natural means safe,” potentially leading to non-evidence-based use without proper medical oversight. Existing research strongly warns of these safety concerns, particularly highlighting the risks of potential herb-drug interactions and the clinical dangers that arise when patients fail to disclose their T&CM use to conventional healthcare providers [1, 3, 4]. Highlighting this informal recommendation culture underscores the necessity for conventional healthcare providers to proactively and routinely inquire about their patients’ T&CM use, fostering an open dialogue to mitigate safety risks and prevent adverse interactions.

In our study, 31.78% of the participants stated that they received information about T&CM modalities from a healthcare professional. Although relying on informal sources remains a prominent concern, this consultation rate with formal medical sources is notably higher than those reported in earlier literature. Rather than being merely a reflection of demographic differences, this relatively high rate may reflect patients’ increasing willingness to consult medical providers and integrate these practices into their conventional care. Indeed, earlier studies conducted both globally and in Türkiye have consistently reported that the rate of receiving T&CM information from health professionals was exceptionally low, often ranging between 4% and 13%, with patients predominantly relying on television, the internet, and personal networks [4, 8, 10, 18]. This apparent tendency of the public to consult healthcare professionals underscores the critical need to equip medical providers with evidence-based T&CM education, enabling them to effectively guide patients and mitigate the risks associated with unsupervised use.

In our study, initial bivariate evaluations indicated that attitudes towards T&CM might differ according to various sociodemographic factors; however, our multiple linear regression model refined this by highlighting increasing age, female gender, marital status, and previous personal experience as the independent predictors of more favorable attitudes, while variables such as education level and chronic disease status did not maintain their significance. This transition from broad bivariate associations to specific independent predictors within our own data mirrors the diversity and inconsistencies found in the broader literature, suggesting that the apparent influence of certain sociodemographic factors may often be confounded by methodological approaches and the specific context of the studied population. Indeed, while population-based studies frequently emphasize female gender and older age as key drivers associated with a higher prevalence of T&CM use and favorable attitudes [19], research conducted on different subgroups often yields contradictory results regarding the impact of variables like marital status, education, and chronic disease [8, 9]. This diversity in findings is likely related to sample differences, methodological variations—such as the lack of multivariate adjustments in some studies—and distinct cultural perceptions of the T&CM concept across communities. Therefore, to resolve these conflicting findings, it would be highly beneficial for future research to evaluate the sociodemographic variables shaping T&CM attitudes not merely in isolation, but through robust multivariate models that account for specific cultural, regional, and contextual dynamics. However, when interpreting these independent predictors, it should be noted that the regression model explains a modest proportion of the variance (R² = 0.095), suggesting that other unmeasured contextual or individual factors also play a significant role in shaping public attitudes.

In our study, participants’ awareness levels were evaluated using a comprehensive list of 25 different T&CM applications. When attempting to contextualize these findings within the broader literature, it becomes evident that there is a significant lack of consensus regarding which interventions strictly fall under the scope of T&CM versus conventional medicine or general wellness. For instance, while some studies assess awareness through a limited number of legally defined practices [8], others evaluate significantly different sets of modalities, and some even classify routine physical activities such as exercise as a T&CM practice [12]. This methodological heterogeneity and the varying number of queried modalities make it highly challenging to directly compare prevalence rates and generalize results across different populations. Consequently, our study underscores that existing literature should be interpreted with caution and highlights the critical need for developing standardized, universally accepted categorizations and measurement tools to accurately evaluate public awareness and utilization of T&CM.

This study has several methodological limitations that should be considered when interpreting the findings. First, the data collection process was conducted online using a convenience sampling approach. Consequently, the sample may not fully represent the general population in Türkiye, as older adults, individuals with limited digital literacy, and those without regular internet access may have been underrepresented. Second, the awareness component of the measurement tool was based solely on participants’ self-reported recognition and familiarity with T&CM terminology. This assessment did not objectively evaluate evidence-based knowledge, clinical understanding, awareness of the scientific evidence, or the safe use of these practices, which constitutes a potential weak validity for this specific component. Third, since the data were entirely self-reported, response bias and social desirability bias may be present despite the use of anonymity and neutral wording. Additionally, although Google Forms™ was configured to allow only one response per Google account to minimize duplicate entries, this possibility cannot be entirely excluded. Moreover, participants may not always have complete or objective information about their family members’ use of T&CM modalities; therefore, these responses should be interpreted as reflecting perceived awareness rather than a verified measure of actual use. Furthermore, the cross-sectional design of the study prevents establishing causal relationships. For instance, there is a potential for reverse causality; while previous T&CM use or recommending it to others may foster favorable attitudes, preexisting favorable attitudes could also drive usage and recommendations. Moreover, a potential non-response bias may exist, as we could not compare the characteristics of those who chose to participate with those who did not. Additionally, no multiple-comparison adjustments were applied to the bivariate analyses, which should be taken into account when interpreting these specific findings. Finally, this study was not developed based on a specific theoretical framework, such as the Health Belief Model or other behavioral theories. Future studies may benefit from theory-driven approaches and more robust, objective measurement tools to better understand the multifaceted factors influencing the awareness, attitudes, and utilization of T&CM practices.

## Conclusions

In conclusion, this study demonstrates that although actual utilization rates of T&CM practices are relatively low, community-dwelling individuals in Türkiye exhibit high awareness of both traditional and modern mind-body modalities, alongside generally favorable attitudes. Furthermore, our multiple linear regression analysis highlights that these favorable attitudes are independently predicted by specific sociodemographic factors, such as older age and female gender, rather than variables like education or chronic disease. The notable discrepancy between low usage or professional consultation rates and high peer recommendation highlights a culture of informal information sharing, which may pose safety risks if left unsupervised. Therefore, conventional healthcare providers must proactively initiate open dialogues with patients regarding T&CM use to mitigate potential adverse interactions. Additionally, recognizing the dual structure of T&CM awareness—spanning from culturally embedded therapies like hijama to global wellness practices like yoga—underscores the need for culturally sensitive and standardized national regulations. Ultimately, these findings provide a crucial basis for promoting the evidence-based, rational use of T&CM and highlight the importance of employing robust multivariate and theory-driven approaches in future research to better understand the complex dynamics shaping public orientation toward these practices.

## Supplementary Information

Supplementary Material 1.

## Data Availability

The datasets supporting the findings of this article are openly available and accessible in an open data repository (Mendeley Data) to ensure transparency and reproducibility. The data can be found using the following Digital Object Identifier https://doi.org/10.17632/bs7d7bngh2.1.

## Abbreviations

T&CM: Traditional and complementary medicine
CACMAS: Complementary, Alternative and Conventional Medicine Attitude Scale
TM: Traditional medicine
CM: Complementary medicine
