# Supplementary material for: Awareness and attitudes toward traditional and complementary medicine among community-dwelling individuals in Türkiye: a cross-sectional online survey
Source: BMC Complement Med Ther. 2026 Jul 22;26:306. doi: 10.1186/s12906-026-05480-6 (PMC13678990; doi:10.1186/s12906-026-05480-6)
Supplement: Supplementary file 1 [file 12906_2026_5480_MOESM1_ESM.docx]

| **Descriptive Information Form** | |
| --- | --- |
| Date of Birth | ….. / ….. / ………. |
| Gender | ☐ Female |
|  | ☐ Male |
| Education Level | ☐ High school and below |
|  | ☐ University and above |
| Marital Status | ☐ Single |
|  | ☐ Married |
| Do you have any chronic diseases? | ☐ Yes |
|  | ☐ No |
| How would you rate your overall health? | ☐ Very good |
|  | ☐ Good |
|  | ☐ Medium |
|  | ☐ Bad |
|  | ☐ Very bad |

| **Knowledge Level Form About Traditional And Complementary Medicine T&CM Practices** | | |
| --- | --- | --- |
|  | I know | I don't know |
| Acupressure | ☐ | ☐ |
| Acupuncture | ☐ | ☐ |
| Ayurveda | ☐ | ☐ |
| Chiropractic | ☐ | ☐ |
| Cupping Therapy | ☐ | ☐ |
| Dry Needling | ☐ | ☐ |
| Energy Medicine (Bioenergy) | ☐ | ☐ |
| Herbal Medicine (Phytotherapy) | ☐ | ☐ |
| Homeopathy | ☐ | ☐ |
| Hydrotherapy | ☐ | ☐ |
| Hypnosis | ☐ | ☐ |
| Lifestyle Diet | ☐ | ☐ |
| Massage | ☐ | ☐ |
| Medicinal Leech Therapy (Hirudotherapy) | ☐ | ☐ |
| Meditation | ☐ | ☐ |
| Music Therapy | ☐ | ☐ |
| Neural Therapy | ☐ | ☐ |
| Nutritional Therapy | ☐ | ☐ |
| Reflexology | ☐ | ☐ |
| Reiki | ☐ | ☐ |
| Relaxation | ☐ | ☐ |
| Tai Chi | ☐ | ☐ |
| Therapeutic Touch | ☐ | ☐ |
| Wet Cupping Therapy (Hijama) | ☐ | ☐ |
| Yoga | ☐ | ☐ |

| **Opinion and Experience Form About Traditional And Complementary Medicine T&CM Practices** | | |
| --- | --- | --- |
|  | Y | N |
| Would you recommend T&CM modalities to your relatives/close ones? | ☐ | ☐ |
| Does anyone in your family use T&CM modalities? | ☐ | ☐ |
| Have you ever used at least one T&CM modality? | ☐ | ☐ |
| Have you ever received information about T&CM modalities from a healthcare professional? | ☐ | ☐ |
| Y: Yes, N: No | | |

| **Complementary, Alternative and Conventional Medicine Attitude Scale (CACMAS)** | | | | | | | | |
| --- | --- | --- | --- | --- | --- | --- | --- | --- |
| Please select the most appropriate one for you (7 corresponding to “I highly agree” and 0 corresponding to “I highly disagree”). | | | | | | | | |
| 1. I was very pleased with the health services provided when I last went to the doctor. | 0 | 1 | 2 | 3 | 4 | 5 | 6 | 7 |
| 2. The human body can heal itself and the duty of healthcare providers is to facilitate this process. | 0 | 1 | 2 | 3 | 4 | 5 | 6 | 7 |
| 3. Spiritual, physical, and mental health should be dealt with as a whole. The person who takes care of my health should also take this into account. | 0 | 1 | 2 | 3 | 4 | 5 | 6 | 7 |
| 4. When I last went to the doctor, I understood the answers given to the important questions I asked about my health. | 0 | 1 | 2 | 3 | 4 | 5 | 6 | 7 |
| 5. Compared to the suggestions and treatments of doctors, I fi nd that the suggestions and treatments of complementary medicine more closely align with my values. | 0 | 1 | 2 | 3 | 4 | 5 | 6 | 7 |
| 6. The positive effect of complementary medicine on health stems from the belief that people are complementary medicine. | 0 | 1 | 2 | 3 | 4 | 5 | 6 | 7 |
| 7. I think complementary healing methods are more natural than the treatments that doctors (modern medicine) use. | 0 | 1 | 2 | 3 | 4 | 5 | 6 | 7 |
| 8. I am very confi dent in the doctor I visit to receive healthcare. | 0 | 1 | 2 | 3 | 4 | 5 | 6 | 7 |
| 9. I think complementary medicine is a threat to public health. | 0 | 1 | 2 | 3 | 4 | 5 | 6 | 7 |
| 10. The health of our body and soul is maintained and sustained by an energy or living force within us. | 0 | 1 | 2 | 3 | 4 | 5 | 6 | 7 |
| 11. I do not trust doctors and hospitals, so I try to go as little as possible. | 0 | 1 | 2 | 3 | 4 | 5 | 6 | 7 |
| 12. Discomfort felt by the patient can be considered as a disruption of the body's balance or impairments in organs that affects the whole body. | 0 | 1 | 2 | 3 | 4 | 5 | 6 | 7 |
| 13. Health and disease are a refl ection of the balance between positive healing energy and negative destructive energy. | 0 | 1 | 2 | 3 | 4 | 5 | 6 | 7 |
| 14. When I last went to the doctor, the doctor did not understand what the problem was. | 0 | 1 | 2 | 3 | 4 | 5 | 6 | 7 |
| 15. A patient’s expectations, health beliefs, and spiritual values should be integrated in healthcare. | 0 | 1 | 2 | 3 | 4 | 5 | 6 | 7 |
| 16. It was diffi cult to talk to my doctor when I last went to the doctor. | 0 | 1 | 2 | 3 | 4 | 5 | 6 | 7 |
| 17. I am often worried that the treatments my doctor suggests will have adverse side effects. | 0 | 1 | 2 | 3 | 4 | 5 | 6 | 7 |
| 18. I feel quite relieved after completing medical treatments or after using complementary medical products. | 0 | 1 | 2 | 3 | 4 | 5 | 6 | 7 |
| 19. I believe that the practice of ambulatory medicine has enabled me to take control of my own health. | 0 | 1 | 2 | 3 | 4 | 5 | 6 | 7 |
| 20. I prefer to treat my health problems on my own. | 0 | 1 | 2 | 3 | 4 | 5 | 6 | 7 |
| 21. Most traditional medical treatment methods activate the healing power that our bodies have. | 0 | 1 | 2 | 3 | 4 | 5 | 6 | 7 |
| 22. Complementary medicine contains ideas and methods that doctors can also benefi t from. | 0 | 1 | 2 | 3 | 4 | 5 | 6 | 7 |
| 23. Treatment approaches not based on scientifi c methods should not be encouraged. | 0 | 1 | 2 | 3 | 4 | 5 | 6 | 7 |
| 24. I believe that complementary medicine for health problems will be more effective than doctors’ recommendations. | 0 | 1 | 2 | 3 | 4 | 5 | 6 | 7 |
| 25. It is important for me to treat a person as a whole when treatment is performed. | 0 | 1 | 2 | 3 | 4 | 5 | 6 | 7 |
| 26. When I last went to the doctor, my treatment was effective. | 0 | 1 | 2 | 3 | 4 | 5 | 6 | 7 |
| 27. When I last went to the doctor, the doctor did not give me enough time. | 0 | 1 | 2 | 3 | 4 | 5 | 6 | 7 |
